# Epicuticular chemistry reinforces the new taxonomic classification of the *Bactrocera dorsalis* species complex (Diptera: Tephritidae, Dacinae)

**DOI:** 10.1371/journal.pone.0184102

**Published:** 2017-09-05

**Authors:** Lucie Vaníčková, Radka Nagy, Antonio Pompeiano, Blanka Kalinová

**Affiliations:** 1 Department of Chemistry and Biochemistry, Faculty of Agronomy, Mendel University in Brno, Brno, Czech Republic; 2 Laboratory of Infochemicals, Institute of Organic Chemistry and Biochemistry, Czech Academy of Sciences, Prague, Czech Republic; 3 Center for Translational Medicine, International Clinical Research Center, St. Anne’s University Hospital, Brno, Czech Republic; 4 Faculty of Forestry and Wood Sciences, Czech University of Life Sciences, Prague, Czech Republic; University of Thessaly School of Agricultural Sciences, GREECE

## Abstract

*Bactrocera invadens* Drew, Tsuruta & White, *Bactrocera papayae* Drew & Hancock, and *Bactrocera philippinensis* Drew & Hancock, key pest species within the *Bactrocera dorsalis* species complex, have been recently synonymized under the name *Bactrocera dorsalis* (Hendel). The closely related *Bactrocera carambolae* Drew & Hancock remains as a discrete taxonomic entity. Although the synonymizations have been accepted by most researchers, debate about the species limits remains. Because of the economic importance of this group of taxa, any new information available to support or deny the synonymizations is valuable. We investigated the chemical epicuticle composition of males and females of *B*. *dorsalis*, *B*. *invadens*, *B*. *papayae*, *B*. *philippinensis*, and *B*. *carambolae* by means of one- and two-dimensional gas chromatography–mass spectrometry, followed by multiple factor analyses and principal component analysis. Clear segregation of complex cuticule profiles of both *B*. *carambolae* sexes from *B*. *dorsalis* (Hendel) was observed. In addition to cuticular hydrocarbons, abundant complex mixtures of sex-specific oxygenated lipids (three fatty acids and 22 fatty acid esters) with so far unknown function were identified in epicuticle extracts from females of all species. The data obtained supports both taxonomic synonymization of *B*. *invadens*, *B*. *papayae*, and *B*. *philippinensis* with *B*. *dorsalis*, as well as the exclusion of *B*. *carambolae* from *B*. *dorsalis*.

## Introduction

The genus *Bactrocera* Macquart (Diptera: Tephritidae) belongs to the family of true fruit flies and contains more than 500 species occurring in South-east Asian and Pacific regions [1]. The *Bactrocera dorsalis* complex comprises nearly 100 species [2,3]. Most species of the complex are of no economic concern. However, the oriental fruit fly, *Bactrocera dorsalis* (Hendel) and closely related species, the Asian papaya fruit fly, *Bactrocera papayae* Drew & Hancock, the Philippine fruit fly, *Bactrocera philippinensis* Drew & Hancock, the carambola fruit fly, *Bactrocera carambolae* Drew & Hancock, and the invasive fruit fly, *Bactrocera invadens* Drew, Tsuruta & White are amongst the world’s most important horticultural pests due to their characteristic polyphagy, high dispersal, and invasiveness [3–6]. While described as separate species, significant debate has occurred in the literature as to whether or not these taxonomic species all constitute separate biological species [6–10]. Recent large-scale multidisciplinary research has failed to detect species-specific differences in a number of their morphological and genetic characteristics, banding patterns of polytene chromosomes, and male pheromone chemoecology [6,7,11]. Lack of consistent morphological discontinuities led to the conclusion that *B*. *papayae* and *B*. *philippinensis* represented a single biological species, with *B*. *philippinensis* synonymized into *B*. *papayae* (hereafter *B*. ‘syn. *philippinensis*’ and *B*. ‘syn. *papayae*’) [10]. Later, integrative taxonomic evidence based on comprehensive investigation of morphology, molecular phylogenetics, cytogenetics, male pheromone, and mating compatibility led to the rejection the taxonomic separation between *B*. ‘syn. *papayae*’, *B*. *invadens* (hereafter *B*. ‘syn. *invadens*’), and *B*. *dorsalis* [6]. Thus, the three previously separate species *B*. ‘syn. *papayae*’, *B*. ‘syn. *philippinensis*’, and *B*. ‘syn. *invadens*’ are now considered a single and biological taxonomic entity, *B*. *dorsalis* [6]. Drew & Romig [9,10], however, have argued against synonymization of *B*. ‘syn. *papayae*’ and *B*. ‘syn. *invadens*’ with *B*. *dorsalis* and insist on keeping them as distinct species, although this in turn has been counter argued [8]. In the case of *B*. *carambolae*, however, morphological, molecular genetic and cytogenetic studies, mating incompatibility, and pheromone differences from *B*. *dorsalis*, *B*. ‘syn. *papayae*’, *B*. ‘syn. *philippinensis*’, and *B*. ‘syn. *invadens*’ have provided sufficient evidence for maintaining *B*. *carambolae* as a discrete taxonomic entity [6,8].

The outermost layer of an insect’s body, the epicuticle, contains a species-specific blend of fatty acid-derived apolar lipids protecting the insect against desiccation [12–14] and pathogens [15]. Cuticular lipids consist of hydrocarbons (CHs), wax esters, sterol esters, ketones, alcohols, aldehydes, and acids. The most prominent cuticular lipids are CHs, occurring in blends of aliphatic or methyl-branched saturated and/or unsaturated hydrocarbons having C_21_ to C_37_ chain lengths with different double bond position(s) and branching(s) [16–18]. Apart from regulating desiccation, the CHs have been secondarily co-opted to serve as insect chemical signals mediating numerous intra- and interspecific interactions [12,18]. In drosophilid flies, these semiochemicals seem to be perceived at relatively short distance by the olfactory organs of the head (antenna and maxillary palps) and/or by contact with the taste organs that are found mostly on the tarsi and proboscis [13,19,20]. Flies show a relatively stable CH profile, although their production can vary through the complete life span and adulthood [13,21,22]. CHs have been proven as excellent chemotaxonomic markers for insect species identification [17,18]. In tephritid fruit flies, the *Anastrepha fraterculus* species complex and the African *Ceratitis* FAR species complex (*C*. *fasciventris*, *C*. *anonae*, *C*. *rosa*) CH profiles have been successfully applied for delimiting putative species [23–26]. Nevertheless, in the *B*. *dorsalis* complex there has been no comprehensive analysis made to date of the CHs for taxonomic distinction of the species.

In tephritid flies, courtship and mating are mediated by multimodal signals including sound, visual, and chemical components. It is generally acknowledged that males initiate mating by means of a sex pheromone that attracts females [27,28]. In the genus *Bactrocera*, however, female-borne pheromones have been reported in *Bactrocera cucurbitae*, *B*. *dorsalis*, and *B*. *oleae* (reviewed in [27] and citations therein). In the *B*. *dorsalis* complex, the male pheromones have been thoroughly investigated to support taxonomic clarification (reviewed in [6,27]). Comprehensive study has not yet been reported, however, of epicuticular compounds in the female *B*. *dorsalis* species complex [29, 30].

To determine how cuticle profiles correspond with recent adjustments in taxonomy of the *B*. *dorsalis* complex and to fill the gap in our knowledge of female chemical ecology in these species, we performed comprehensive gas chromatography–mass spectrometry (GC/MS) and two-dimensional gas chromatography–mass spectrometry (GC×GC/MS) analyses of four geographically different but genetically similar *Bactrocera dorsalis* complex species formerly classified as *B*. *dorsalis*, *B*. ‘syn. *papayae*’, *B*. ‘syn. *philippinensis*’, and *B*. ‘syn. *invadens*’, and compared them with the genetically distinct *B*. *carambolae*. While recognizing that the current nomenclature position is that only *B*. *dorsalis* and *B*. *carambolae* are valid taxonomic names [8], this paper continues to use the additional old names of *B*. ‘syn. *invadens*’, *B*. ‘syn. *papayae*’, and *B*. ‘syn. *philippinensis*’ to allow direct comparison with pre-2015 literature on the *B*. *dorsalis* species complex.

## Materials and methods

### Flies

All flies were obtained in 2011 from the Insect Pest Control Laboratory (IPCL) of the Food and Agriculture Organization/International Atomic Energy Agency (FAO/IAEA, Seibersdorf, Austria), where they had been reared under standard laboratory conditions on the standard wheat-based artificial diet for *Bactrocera* spp. [31] at 25°C, 65% RH, and photoperiod of 12:12 [L:D] h. The *B*. *carambolae* fruit flies had originated from Paramaribo, Suriname, from star fruit *Averrhoa carambola*. They had been reared at IPCL since 2010. Flies of the F6 generation were used. *Bactrocera* ‘syn. *philippinensis*’ originated from Guimaras Island, Philippines, from papaya *Carica papaya*. They had been reared at the IPCL from 2010 and the F5 generation was tested. *Bactrocera* ‘syn. *papayae*’ had originated from Serdang, Malaysia, from banana *Musa* sp. The insect had been at the IPCL since 2010 and the F5 generation was used. *Bactrocera* ‘syn. *invadens*’ had originated at a laboratory colony from Kenya kept at IPCL from 2009 and the F20 generation was tested. *Bactrocera dorsalis* had originated from Saraburi, Thailand, from mango *Mangifera indica*, was at the IPCL from 2010, and the F6 generation was extracted. Pupae of the five fruit fly entities were sent from the IPCL to the Institute of Organic Chemistry and Biochemistry (IOCB), Czech Academy of Sciences in 2011. At the IOCB, pupae were kept separately in glass containers (30 × 30 × 30 cm) under conditions of temperature 25°C, 65% RH, and photoperiod 12:12 [L:D] h. Newly hatched flies were sexed, kept separately, and provided with water and artificial diet consisting of sugar cane:yeast (3:1). The taxonomic classification of *B*. *dorsalis* (Saraburi, Thailand) was kindly provided by RAI Drew (Griffith University, Brisbane, Australia). The remaining flies were taxonomically determined by MK Schutze (Queensland University of Technology, Brisbane, Australia).

Mature flies 9 days old were used for experimentation. Flies were chilled for 5 min prior to cuticle lipid extraction. Males and females were individually placed in 2 ml glass vials, covered by 200 μl of distilled *n*-hexane (Sigma-Aldrich, Czech Republic) containing 5 ng/μl of 1-bromundecane (Fluka, Czech Republic) as an internal standard. After 5 min, the solvent was transferred to another vial, closed, and then stored in a freezer until used for analyses. Five males and five females from each entity were used.

### Chemical analysis

The initial screening for determination of differences and/or similarities in the cuticle profiles of *Bactrocera* spp. was performed with a Hewlett Packard HP 6890 GC System connected to a Hewlett Packard 5973 Mass Selective Detector. For analyses, an HP-5MS capillary column (30 m × 250 μm i.d. × 0.25 μm film; Agilent Technologies, Santa Clara, CA, USA) was used. Temperature was programmed from 150°C to 300°C at a rate of 5°C/min with 10 min final hold at 320°C. Samples (1 μl) were injected using a splitless mode with He as the carrier gas (constant pressure, 1 ml/min). Electron ionization at 70 eV was used in the range from 25 to 600 *m/z*, with ion source temperature 250°C and quadrupole temperature 150°C.

Detailed identification and quantification of the components was performed by GC×GC/MS, using a LECO Pegasus 4D instrument (LECO Corp., St. Joseph, MI, USA) equipped with a non-moving quad-jet cryomodulator connecting the 1st and the 2nd columns. The methodology has been described in detail elsewhere [23,25,26]. A series of *n*-alkanes (C_12_–C_40_; Sigma-Aldrich) was used to determine the retention indices for the analytes. The compounds were identified by a comparison of their MS fragmentation patterns and retention indices with values published previously [22,23,26,29,32,33].

### Statistical evaluation

The relative peak areas of 59 compounds (as identified by GC×GC/MS in the deconvoluted total ion chromatogram mode) were calculated for each replicate of the entities study. Differences in chemical composition of the samples from study entities were analysed by multiple factor analyses (MFA) and/or principal component analysis (PCA). Prior to MFA and/or PCA, peak areas were transformed logarithmically, intraspecific scaling was performed by dividing each species score by its standard deviation, and the data were centred by species’ scores. In PCA analyses, hierarchical clustering based on Pearson correlation showed that entities with similar chemical profiles cluster together. A heat map was used to visualize compounds organized as matrices [23]. Dendrograms were created using correlation-based distances and the Ward method of agglomeration was used in the analysis [34]. To further examine differences between the five female entities, the percentage contribution of each female-specific compound (fatty acid esters) to the average dissimilarity between female entities was calculated with similarity percentage analysis (SIMPER) [35]. All computations were performed with R 3.1.2 [36], and the R packages FactoMineR [37] and gplots [38] were used.

## Results

**Fig 1:**
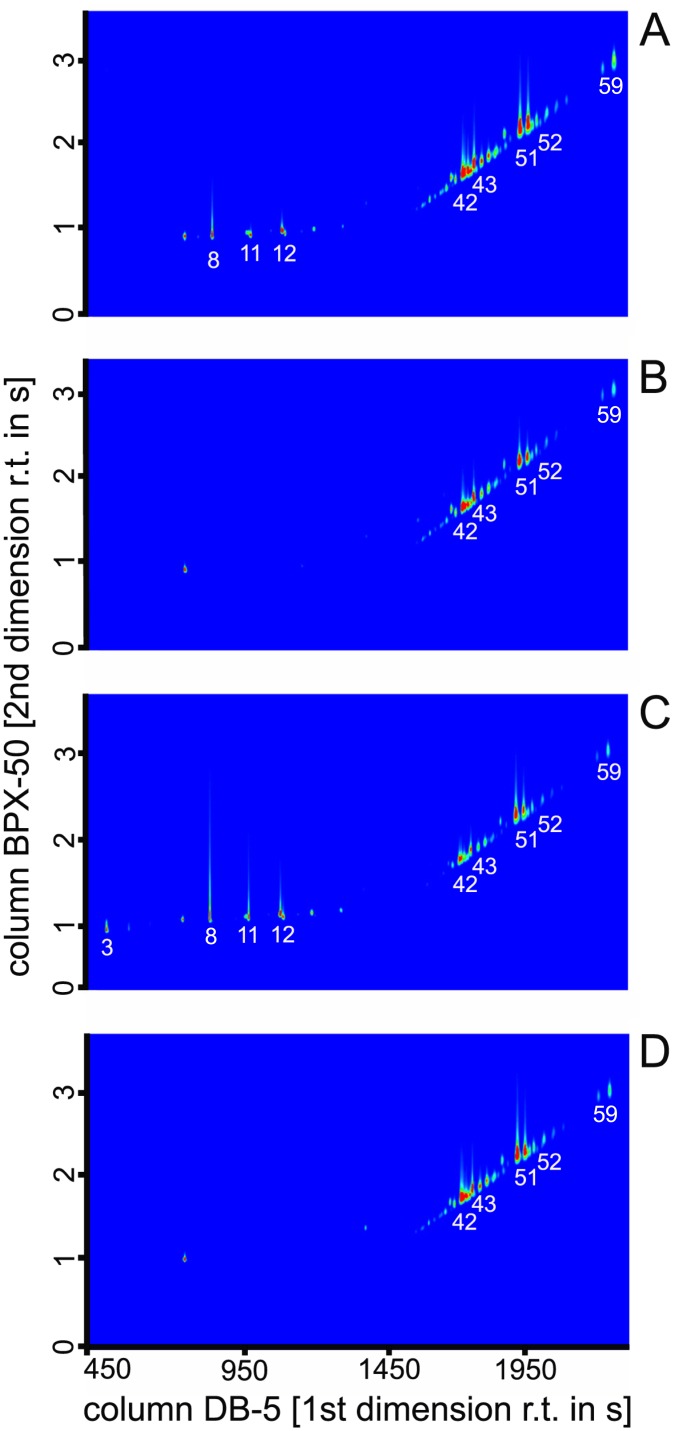
Two-dimensional chromatogram of GC×GC/MS analysis of *Bactrocera dorsalis* and *B*. *carambolae* cuticular profiles. Cuticular profiles of *B*. *carambolae* female (A) and male (B) and of *B*. *dorsalis* female (C), male (D). Intensity of the signals is colour coded from blue (zero) to red (maximum). The compounds are assigned according to S1 Table.

In total, 59 compounds were identified in hexane body washes of males and females of *B*. *carambolae*, *B*. *dorsalis*, *B*. ‘syn. *invadens*’, *B*. ‘syn. *papayae*’, and *B*. ‘syn. *philippinensis*’. The compounds comprised a complex mixture of three fatty acids, 22 esters, as well as 32 hydrocarbons and two aldehydes with chain lengths ranging from 12 to 40 carbons (Fig 1, S1 Fig, S1 Table).

**Fig 2:**
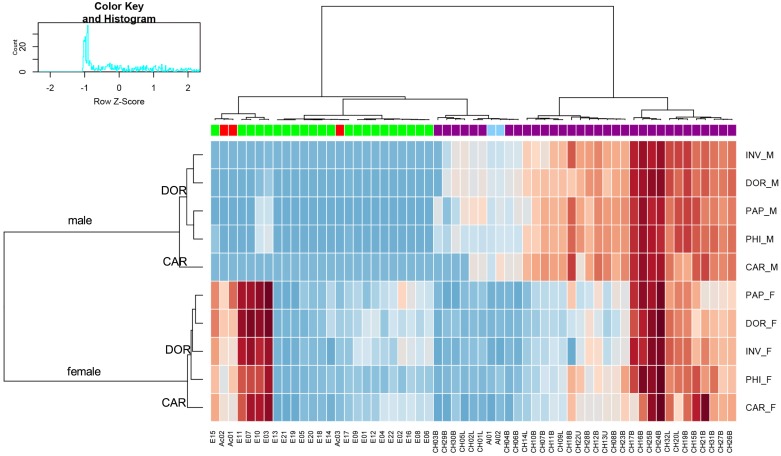
Heat map of all 59 compounds and the five *Bactrocera* entities from the GC×GC/MS data set. M = male, F = female, DOR = *B*. *dorsalis*, CAR = *B*. *carambolae*, INV = *B*. ‘syn. *invadens*’, PAP = *B*. ‘syn. *papayae*’, PHI = *B*. ‘syn. *philippinensis*’. Columns are colour coded according to chemical classes (i.e. red = fatty acid Ac01–03, green = fatty acid ester E01–22, violet = cuticular hydrocarbon CH01–32, blue = aldehyde Al01–02). The dendrograms are created using correlation-based distances and the Ward method of hierarchical clustering (*P* < 0.05). The compounds are assigned according to S1 Table.

**Fig 3:**
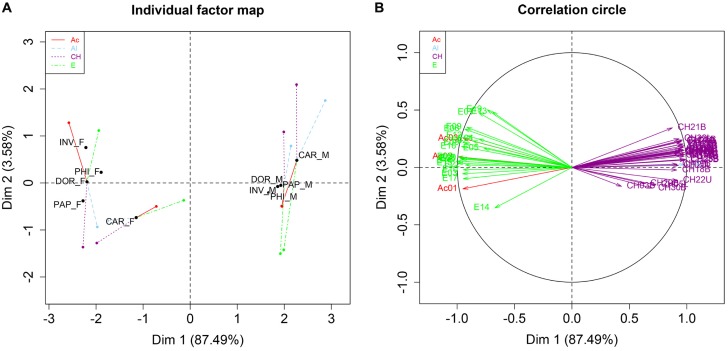
MFA of transformed GC×GC/MS data of 59 compounds identified in five *Bactrocera* entities. M = male, F = female, DOR = *B*. *dorsalis*, CAR = *B*. *carambolae*, INV = *B*. ‘syn. *invadens*’, PAP = *B*. ‘syn. *papayae*’, PHI = *B*. ‘syn. *philippinensis*’. (A) Score plot describing the species and chemical class modalities of the first two factors, (B) projection of variables onto the plane defined by the first two principal components of the MFA. The coordinates for each variable are the correlation coefficients with the two first principal components (red Ac01–03 = fatty acid, blue Al01–02 = aldehyde, violet CH01–32 = cuticular hydrocarbon, green E01–22 = fatty acid ester). The compounds are assigned according to S1 Table.

Quantitative and qualitative differences were found in male versus female chemical profiles among the investigated entities when applying GC/MS. From the chemical mixture, 32 hydrocarbons were common for both sexes, whereas 22 fatty acid esters and three fatty acids were specific for females (Fig 1, S1 Fig). GC×GC/MS allowed for detailed structural identification of all compounds present in the cuticle extracts. A heat map was constructed to visualize the relative proportions of all 59 compounds in each of the entities studied (Fig 2, S1 Table). In the compound dendrogram, the majority of cuticular hydrocarbons formed one cluster, while fatty acids, esters and aldehydes together with several CHs grouped into a second main cluster. The three most abundant compounds among all the entities were methyl-branched hydrocarbons with 32-, 34- and 36-carbon backbones (CH16B, CH17B, CH24B, CH25B). A mixture of 3,9-/3,7-dimethyl C_31_ (CH18B) was the most variable across the males and females of all entities studied. Ethyl dodecanoate (E03), ethyl tetradecanoate (F07), ethyl (*Z*)-hexadec-9-enoate (E10), ethyl hexadecanoate (E11), ethyl (*Z*)-octadec-9-enoate (E15), and two acids (myristoleic acid Ac01, gondoic acid Ac02) were compounds most abundant in females (Fig 2, S1 Table). Distinct sex specificity of the analysed chemical profiles of males and females was clearly visible. *Bactrocera carambolae* males and females were distinctly separated from the remaining entities of *B*. *dorsalis* (Fig 2). The separation of sexes and species based on the complex chemical profiles of the cuticle was further supported by MFA (Fig 3A and 3B). In the individual factor map (Fig 3A), where the first two axes represent 91.1% of the total variance, females were distinctively separated from males along the first axis (Dim 1). Within both sexes, *B*. *carambolae* formed groups separated from those of the remaining entities studied (Dim 2). *Bactrocera* spp. males were separated according to their specific CH profiles, while females were characterized by esters and fatty acids (Fig 3B). Upon closer examination, cuticular hydrocarbons and aldehydes are seen to be correlated with formation of a *B*. *carambolae* male cluster and its separation from *B*. *dorsalis*, whereas fatty acids and esters are correlated with formation of the *B*. *carambolae* female group.

**Fig 4:**
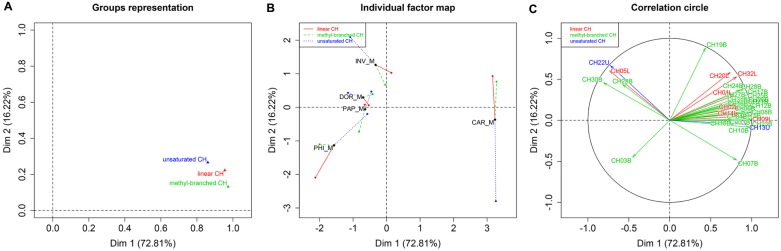
MFA of transformed GC×GC/MS data of 32 cuticular hydrocarbons identified in five *Bactrocera* entities. M = male, DOR = *B*. *dorsalis*, CAR = *B*. *carambolae*, INV = *B*. ‘syn. *invadens*’, PAP = *B*. ‘syn. *papayae*’, PHI = *B*. ‘syn. *philippinensis*’. (A) Representation of groups of variables, (B) score plot describing the species and chemical class modalities of the first two factors, (C) projection of variables onto the plane defined by the first two principal components of the MFA. The coordinates of each variable are the correlation coefficients for the two first principal components (linear CH = red, methyl-branched CH = green, unsaturated CH = blue). The compounds are assigned according to S1 Table.

MFA was applied for the evaluation of CHs as chemotaxonomic markers for species differentiation in males (Fig 4). The first two dimensions of the MFA axes represented 89% of the total variance. Linear and methyl-branched CH weighted most heavily Dim 1 (Fig 4A). Different conclusions can be drawn regarding the contribution of each group of variables to axis 2. The contribution of unsaturated CHs appears to be statistically most significant when compared to the low contribution of the other variables (linear and methyl-branched CHs), which are the least useful groups of compounds for discriminating among the species. *Bactrocera carambolae* was separated from *B*. *dorsalis* (i.e. *B*. *dorsalis*, *B*. ‘syn. *invadens*’, *B*. ‘syn. *papayae*’, and *B*. ‘syn. *philippinensis*’) along the first axis (Dim 1), indicating that it is possible to distinguish these two species base on their CH profiles. Methyl-branched CHs were positively correlated with *B*. *carambolae*, while unsaturated hydrocarbon (CH22U) tritriacontene was positively correlated with formation of the *B*. ‘syn. *invadens*’ males group (Fig 4C). Separate heat maps for male and female CH profiles were constructed (S2 Fig). In males, there was a clear segregation of *B*. *carambolae* from the remaining species complex entities. In females, however, *B*. *carambolae* females clustered together with *B*. *dorsalis* and *B*. ‘syn. *philippinensis*’ (S2 Fig), thus indicating that CH profiles of females are not species- specific.

The PCA of female-specific fatty acid esters revealed the formation of four distinct clusters, where *B*. *dorsalis* grouped together with *B*. ‘syn. *philippinensis*’, whereas *B*. ‘syn. *invadens*’, *B*. *carambolae*, and *B*. ‘syn. *papayae*’ formed another three separated clusters (S3 Fig). The first two dimensions represented 70% of the total variance. Ethyl (*Z*)-octadec-9-enoate (E15), ethyl pentacosanoate (E21), ethyl docosanoate (E18), and ethyl eicosanoate (E17) were the most important compounds positively correlated with formation of the *B*. *dorsalis* and *B*. ‘syn. *philippinensis*’ group, whereas ethyl octadecanoate (E16) and methyl (*Z*)-tetradec-9-enoate (E05) were negatively correlated with *B*. ‘syn. *invadens*’, *B*. *carambolae*, and *B*. ‘syn. *papayae*’ clustering (S3 Fig). For each pairwise female comparison, SIMPER (S2 Table) allowed identification of the first 10 esters contributing most to the differentiation between the four female entities of the *B*. *dorsalis* complex and the *B*. *carambolae* females. Ethyl decanoate (E01), ethyl dodecanoate (E03), and ethyl tetradecanoate (E07) contributed most to the overall dissimilarity.

## Discussion

Our analyses of the complex mixture of cuticle blends allowed for characterizing species- and sex-specific cuticle profiles in *B*. *carambolae* and *B*. *dorsalis*. This supports the taxonomic synonymizations within the *B*. *dorsalis* complex [6,8] and for keeping *B*. *carambolae* as a separate species. The existence of morphologically similar biological entities within the *B*. *dorsalis* species complex had been recognized for more than 40 years [39]. Several taxonomic revisions of the group have attempted to clarify the taxonomic relationships without reaching conclusive results [40–42]. Lack of morphological discontinuities resulted in first synonymization of *B*. ‘syn. *philippinensis*’ with *B*. ‘syn. *papayae*’ [10]. Later, integrative taxonomic evidence based on morphological, genetic, chemoecological, and behavioural studies led to synonymization of *B*. ‘syn. *invadens*’ and *B*. ‘syn. *papayae*’ with *B*. *dorsalis* [11,43]. *Bactrocera carambolae* has continued to be regarded as a distinct species due to subtle but reliable differences in morphology, molecular genetics, chemoecology, and behaviour [6]. Nevertheless, Drew & Romig [9,10] argue against synonymization of *B*. ‘syn. *papayae*’ and *B*. ‘syn. *invadens*’ with *B*. *dorsalis* and insist on keeping them as two distinct species [9,10], although this has been countered [8]. Our data do not agree with Drew & Romig [9,10] but rather show no species specificity in cuticular profiles of *B*. *dorsalis*, and the former *B*. ‘syn. *invadens*’, *B*. ‘syn. *papayae*’, and *B*. ‘syn. *philippinensis*’. While separating males of *B*. *carambolae* from *B*. *dorsalis sensu lato* (*i*.*e*. *B*. *dorsalis*, *B*. ‘syn. *invadens*’, *B*. ‘syn. *papayae*’, and *B*. ‘syn. *philippinensis*’), our data further prove that epicuticular profiles are reliable chemotaxonomic markers for species delimitation. Hydrocarbons have been used for species identification in many insect orders, including the Hemiptera [44,45], Hymenoptera [46–48], Isoptera [49,50], Orthoptera [51], and Diptera [18,52,53]. In fruit flies, CHs have been successfully used for species and population discrimination within the South American fruit fly species complex and the so-called African *Ceratitis* FAR complex species (*C*. *fasciventris*, *C*. *anonae*, *C*. *rosa*) [22–26]. Though CH analysis followed by PCA or MFA is widely used today, its scope and limits still require validation using multiple samples from large geographic areas [23]. It is well documented that diet and environmental condition also influence the CH composition in flies [54–56]. Entities of *B*. *dorsalis* and *B*. *carambolae* investigated in the present study were all reared through several generations under identical laboratory conditions and diet. The fact that the chemical profiles of males remain diverse in the case of *B*. *carambolae* compared to *B*. *dorsalis* indicates that they are heritable and genetically conserved, as previously reported for other fruit fly species [23,57,58]. On the other hand, it was not possible to differentiate females of *B*. *dorsalis* and *B*. *carambolae* using only the CH profiles, thus underscoring a limited applicability of CHs for *Bactrocera* female chemotaxonomy.

We determined that the chemical profiles consisting of mixtures of aliphatic hydrocarbons, fatty acid esters, fatty acids, and aldehydes were sex-specific and differed both qualitatively and quantitatively. As reviewed by Schutze et al. [6], previous chemoecological studies had focused mainly on comparing chemistry of male rectal glands from *B*. *dorsalis sensu lato* and *B*. *carambolae*. No spiroundecanes—previously reported in the aeration extracts of *B*. *dorsalis* females [59]–were identified here. That no spiroundecanes were found among *B*. *dorsalis* female-specific compounds may possibly be due to differences in the analytical techniques used (aeration in previous experiments *versus* solvent washes in our experiments). Our findings are in agreement with Goh et al. [29], who chemically characterized cuticular washes of Malaysian *B*. *dorsalis* without detecting spiroundecanes in females. In contrast with the aforementioned studies, we did identify 22 female-specific fatty acid esters (FEs) present in all investigated species. Due to the short extraction time, the detected FEs should not have been extracted from inner organ systems but rather from the cuticular surface. Fatty acid esters and free fatty acids have been established as cuticular components in many insect species [28]. They usually occur in homologous series of even carbon numbers, in most cases ranging between 10 and 36 carbon atoms [60]. From 22 fatty acid esters identified here in epicuticular washes of *B*. *dorsalis sensu lato* and *B*. *carambolae*, nine esters (i.e. ethyl decanoate, methyl dodecanoate, ethyl dodecanoate, methyl tetradecanoate, ethyl tetradecanoate, methyl (*Z*)-hexadec-9-enoate, methyl hexadecanoate, ethyl hexadecanoate, and ethyl (*Z*)-octadec-9-enoate) were recently identified in female sex pheromone of *B*. *oleae* [30]. This capacity of *B*. *oleae* females to secrete and release the pheromone is unique among tephritid fruit flies, as this is generally the task of the males. The origin and function of FEs in *B*. *dorsalis* and *B*. *carambolae* females are not known. We can only guess about the possible significance of this finding in relation to the biology of females. Although flies of *Bactrocera* do not mark fruit, females appear to be capable of recognizing and avoiding oviposition in occupied fruit [61]. Some of the identified compounds may contribute to this recognition. Canale et al. [30] suggested that some of the FEs, namely electrophysiologically and behaviourally active ethyl decanoate, may be involved in female–female aggression over single oviposition sites, as was recently described in the case of the olive fruit fly [62,63]. Furthermore, the female FEs may serve as conspecific identification cues for mating males. Methyl hexadecanoate, a component of *B*. *oleae* female rectal glands, showed dose-dependent attraction of virgin males [30]. However a lack of species specificity in their composition contradicts the latter-mentioned possibility. Future studies will be conducted in order to shed more light on the possible role of FEs in the chemical communication by *B*. *dorsalis* and *B*. *carambolae*.

Free fatty acids (FAs) were identified here for the first time as a part of tephritid cuticle. In insects, FAs are responsible for resistance to fungal infection [64]. Cuticular FAs are toxic and fungistatic but may also be stimulatory [65]. Differences in FAs chemical composition among insects may be responsible for susceptibility or resistance to fungal infection. Determination of the FAs found in cuticular lipids can contribute substantially to the knowledge concerning insect defence mechanisms [66]. In addition, due to their intrinsic antibacterial effect, fatty acids are suggested to produce the non-fouling characteristics of the cuticle surface [67]. There is a gap in our understanding as to the function of FAs present on *B*. *dorsalis* and *B*. *carambolae* female cuticle. Detailed research in this field is therefore greatly encouraged, such as would be focused upon resistance of *Bactrocera* to entomopathogenic fungi or bacteria.

Overall, these analyses of cuticular profiles of five *Bactrocera* entities demonstrated that the complex cuticular blends are species-specific when comparing *B*. *carambolae* with *B*. *dorsalis sensu lato*, thus supporting the current synonymization of key species within the *B*. *dorsalis* complex and keeping *B*. *carambolae* as a separate species. Cuticular hydrocarbons were proven to be reliable chemotaxonomic markers for distinguishing *B*. *carambolae* and *B*. *dorsalis* males, but this was not confirmed for females of the same species. Furthermore, female-specific fatty acid esters were not effective for the separation of *B*. *carambolae* from *B*. *dorsalis*. The possibility that these compounds might nevertheless be involved in the chemical communication of *B*. *dorsalis* points to a future direction for our research.

## Supporting information

S1 Table Relative contribution (%) of compounds identified in the cuticular hexane body washes of males and females of *Bactrocera carambolae* and *B*. *dorsalis* (*B*. *dorsalis*, *B*. ‘syn. *papayae*’, *B*. ‘syn. *philippinensis*’, *B*. ‘syn. *invadens*’). (PDF)

S2 Table Comparison of the average abundance of important fatty acid esters between five entities of *Bactrocera* (*B*. *dorsalis*, *B*. *carambolae*, *B*. ‘syn. *invadens*’, *B*. ‘syn. *papayae*’, *B*. ‘syn. *philippinensis*’). Compounds are listed in order of their contributions (δ_i_) to the average dissimilarity 5(δ_i_) between the two groups, with a cut-off when the cumulative percent contribution (∑δ_i_%) to δ_i_ reaches 70%. Numbering of the compounds corresponds to Fig 1 and S1 Table.
(PDF)

S1 Fig Two-dimensional chromatogram of GC×GC/MS analysis of *Bactrocera spp*. cuticular profiles. Cuticular profiles of female (A) and male (B). Intensity of the signals is colour coded from blue (zero) to red (maximum).
(PDF)

S2 Fig Heat maps of all 32 cuticular hydrocarbons and five *Bactrocera* entities from GC×GC/MS data set. (A) male, (B) female. DOR = *B*. *dorsalis*, CAR = *B*. *carambolae*, INV = *B*. ‘syn. *invadens*’, PAP = *B*. ‘syn. *papayae*’, PHI = *B*. ‘syn. *philippinensis*’). Columns are colour coded according to chemical classes (violet CHL = linear hydrocarbon, red CHB = methyl-branched hydrocarbon, green CHU = unsaturated hydrocarbon). Dendrograms are created using correlation-based distances and the Ward method of hierarchical clustering (*P* < 0.05). Compounds are assigned according to S1 Table.
(PDF)

S3 Fig Principal component analyses (PCA) of transformed GC×GC/MS data of 22 female-specific fatty acid esters. E01–22 were identified on the cuticle of five *Bactrocera* entities (DOR = *B*. *dorsalis*, CAR = *B*. *carambolae*, INV = *B*. ‘syn. *invadens*’, PAP = *B*. ‘syn. *papayae*’, PHI = *B*. ‘syn. *philippinensis*’). (A) Variables factor map represents projection of variables on plane defined by the first two principal components. (B) Hierarchical clustering is score plot describing the species and their clustering. Colours indicate particular clusters.
(PDF)
