# Supplementary material for: Epicuticular chemistry reinforces the new taxonomic classification of the Bactrocera dorsalis species complex (Diptera: Tephritidae, Dacinae)
Source: PLoS One. 2017 Sep 5;12(9):e0184102. doi: 10.1371/journal.pone.0184102 (PMC5584755; doi:10.1371/journal.pone.0184102)
Supplement: S1 Table — (PDF) [file pone.0184102.s001.pdf]

Table 1 Relative contribution (%) of compounds identified in the cuticular hexane body washes of males and females of *Bactrocera carambolae* and *B. dorsalis* (*B. dorsalis, B. papayae, B. philippinensis, B. invadens*).

No.	Abbrev.[a]	Compound	RI[b]	*B. carambolae*		*B. dorsalis*		*B. invadens*		*B. papayae*		*B. philippinensis*	
				female	male	female	male	female	male	female	male	female	male
1	E01	Ethyl decanoate	1396	0.08±0.01	-	0.30±0.12	-	0.22±0.08	-	0.05±0.01	-	0.04±0.01	-
2	E02	Methyl dodecanoate	1523	0.18±0.02	-	0.19±0.09	-	0.36±0.01	-	0.77±0.18	-	0.05±0.02	-
3	E03	Ethyl dodecanoate	1590	11.60±1.3	-	12.91±1.34	Tr	11.95±1.78	-	13.70±0.32	0.04±0.01	8.94±0.69	0.08±0.01
4	E04	Ethyl tridecanoate	1664	0.24±0.02	-	0.05±0.01	-	0.11±0.04	-	0.21±0.01	-	0.03±0.01	-
5	E05	Methyl (*Z*)-tetradec-9-enoate	1708	0.02±0.01	-	Tr	-	0.03±0.01	-	0.08±0.01	-	Tr	-
6	E06	Methyl tetradecanoate	1726	0.16±0.03	-	0.26±0.01	-	0.28±0.05	-	0.25±0.02	-	0.09±0.02	-
7	Ac01	(*Z*)-Tetradec-9-enoic acid	1776	0,52±0.04	-	1.89±0.14	-	1.00±0.03	-	2.97±0.14	-	1.13±0.12	-
8	E07	Ethyl tetradecanoate	1795	8.21±0.51	-	11.08±1.74	-	10.03±0.41	-	8.16±0.23	-	4.76±0.12	-
9	E08	Methyl (*Z*)-hexadec-9-enoate	1908	0.14±0.02	-	0.16±0.01	-	0.19±0.01	-	0.29±0.02	-	0.10±0.04	-
10	E09	Methyl hexadecanoate	1928	0.08±0.01	-	0.16±0.04	-	0.20±0.02	-	0.16±0.01	-	0.08±0.03	-
11	E10	Ethyl (*Z*)-hexadec-9-enoate	1975	7.24±1.02	-	9.91±1.30	-	6.53±0.12	-	10.11±0.45	0.04±0.01	4.71±0.12	0.06±0.02
12	E11	Ethyl hexadecanoate	1988	3.33±0.01	-	9.12±0.42	-	7.49±0.23	-	7.88±0.41	-	4.27±0.14	-
13	E12	Ethyl (*Z*)-heptadec-9-enoate	2071	0.06±0.01	-	0.29±0.06	-	0.13±0.02	-	0.13±0.02	-	0.03±0.01	-

14	E13	Methyl octadecanoate	2130	-	-	-	-	Tr	-	Tr	-	-	-
15	E14	Ethyl (9*Z*,12*Z*)-octadeca-9,12-dienoate	2166	tr	-	0.16±0.02	-	-	-	0.17±0.01	-	0.09±0.01	-
16	E15	Ethyl (*Z*)-octadec-9-enoate	2173	1.28±0.21	-	2.50±0.12	-	1.50±0.04	-	2.24±0.14	-	1.25±0.23	Tr
17	E16	Ethyl octadecanoate	2195	0.24±0.03	-	0.44±0.02	-	0.27±0.08	-	0.43±0.12	-	0.20±0.04	-
18	Ac02	(*Z*)-Icos-11-enoic acid	2377	0.47±0.12	-	1.00±0.05	-	0.53±0.12	-	0.79±0.06	-	0.42±0.06	-
19	E17	Ethyl eicosanoate	2394	0.05±0.01	-	0.12±0.07	-	0.04±0.01	-	0.12±0.01	-	0.08±0.01	-
20	Ac03	(*Z*)-Docos-13-enoic acid	2575	0.04±0.02	-	0.11±0.07	-	0.05±0.02	-	0.05±0.01	-	0.03±0.01	-
21	E18	Ethyl docosanoate	2593	0.03±0.01	-	0.04±0.02	-	0.02±0.01	-	0.05±0.02	-	0.03±0.01	-
22	CH01L	*n*-Hexacosane	2602	Tr	0.04±0.01	Tr	0.07±0.00	0.04±0.01	0.05±0.01	0.06±0.00	0.19±0.01	0.02±0.00	0.04±0.02
23	CH02L	*n*-Heptacosane	2702	Tr	0.05±0.02	0.02±0.01	0.05±0.01	0.03±0.01	0.04±0.01	0.05±0.00	0.17±0.02	0.03±0.01	0.05±0.01
24	CH03B	11-/13-MeC_{27}	2736	-	-	-	-	-	-	0.03±0.00	0.07±0.01	-	0.04±0.00
25	CH04B	3-MeC_{27}	2769	Tr	0.04±0.01	Tr	0.05±0.01	0.02±0.00	0.03±0.01	0.02±0.01	0.07±0.02	Tr	0.06±0.01
26	E19	Tetradecyl dodecanoate	2769	-	-	Tr	-	Tr	0.00	-	-	Tr	-
27	E20	Ethyl tetracosanoate	2794	0.02±0.01	-	0.04±0.01	-	0.02±0.01	-	0.05±0.02	-	0.02±0.01	-
28	CH05L	*n*-Octacosane	2802	-	-	Tr	0.08±0.02	0.03±0.01	0.08±0.02	0.04±0.00	0.13±0.02	0.02±0.00	0.04±0.00
29	Al01	Hexacosanal	2841	-	0.03±0.01	Tr	0.04±0.01	-	0.02±0.01	Tr	0.03±0.01	0.04±0.02	0.05±0.02

30	CH06B	2-MeC_{28}	2864	0.06±0.02	0.05±0.01	Tr	0.08±0.02	Tr	0.03±0.01	-	0.08±0.01	0.04±0.01	0.05±0.01
31	E21	Ethyl pentacosanoate	2895	-	-	Tr	-	-	-	-	-	-	-
32	CH07B	11-/13-MeC_{29}	2935	0.15±0.02	0.86±0.03	0.15±0.03	0.36±0.12	0.12±0.03	0.23±0.17	0.18±0.04	0.68±0.11	0.37±0.03	0.84±0.03
33	CH08B	3-MeC_{29}	2976	0.38±0.03	0.42±0.04	0.18±0.02	0.60±0.01	0.28±0.02	0.51±0.03	0.48±0.03	1.00±0.05	0.66±0.24	1.13±0.04
34	E22	Ethyl hexacosanoate	2996	0.25±0.11	-	0.28±0.03	-	0.08±0.03	-	0.25±0.02	-	0.13±0.01	-
35	CH09L	*n*-Triacontane	3002	0.18±0.05	0.64±0.12	0.11±0.01	0.32±0.07	0.15±0.03	0.36±0.04	0.20±0.06	0.62±0.04	0.35±0.02	0.69±0.02
36	CH10B	12-MeC_{30}	3036	0.15±0.03	0.50±0.06	0.07±0.01	0.26±0.01	0.13±0.04	0.19±0.02	0.16±0.03	0.32±0.05	0.17±0.06	0.34±0.09
37	CH11B	6-MeC_{30}	3049	0.22±0.02	0.61±0.04	0.14±0.02	0.31±0.02	0.21±0.05	0.34±0.03	0.22±0.04	0.55±0.05	0.26±0.03	0.68±0.01
38	Al02	Octacosanal	3056	0.02±0.01	0.09±0.01	0.02±0.00	0.04±0.01	-	0.02±0.00	0.02±0.00	0.06±0.00	0.08±0.01	0.06±0.02
39	CH12B	4-MeC_{30}	3069	0.47±0.04	2.02±0.39	0.29±0.04	1.07±0.28	0.55±0.16	1.18±0.03	0.60±0.01	1.53±0.32	0.79±0.10	1.53±0.12
40	CH13U	$C_{31:1}$	3096	0.39±0.06	1.25±0.13	0.10±0.04	0.30±0.03	0.15±0.02	0.54±0.04	0.19±0.05	1.10±0.31	0.64±0.03	1.86±0.07
41	CH14L	*n*-Hentriacontane	3103	0.08±0.01	0.32±0.07	0.05±0.01	0.21±0.05	0.07±0.03	0.21±0.02	0.05±0.01	0.17±0.02	0.12±0.03	0.33±0.04
42	CH15B	11-/13-MeC_{31}	3123	7.66±1.56	3.24±0.52	0.70±0.22	1.84±0.44	1.04±0.03	1.94±0.02	1.42±0.03	5.31±0.27	4.30±0.05	4.33±0.12
43	CH16B	13,23-/11,21-/11,23-DiMeC_{30}	3163	5.68±0.45	26.78±0.56	6.35±0.63	15.90±1.56	6.41±1.32	19.22±1.02	9.82±0.88	24.01±1.21	12.24±1.20	20.85±1.02
44	CH17B	5,13-DiMeC_{30}	3189	1.66±0.11	9.39±0.54	4.01±0.33	10.25±1.83	7.52±0.26	10.64±1.02	5.83±0.92	8.45±0.19	4.87±0.87	9.38±0.41
45	CH18B	3,9-/3,7-DiMeC_{31}	3209	2.47±0.42	6.13±0.41	0.38±0.01	1.21±0.21	-	3.30±0.21	0.97±0.12	3.61±0.32	1.44±0.12	3.19±1.02

46	CH19B	8,10-/8,12-DiMeC_{31}	3270	2.64±0.36	0.44±0.03	2.14±0.49	5.45±0.35	3.04±0.31	5.97±0.36	2.35±0.32	6.44±0.42	3.32±0.06	6.97±0.21
47	CH20L	*n*-Tritriacontane	3303	0.64±0.48	0.59±0.05	2.28±0.41	3.44±0.15	2.86±0.12	5.04±0.75	2.37±0.12	4.91±0.43	2.98±0.01	5.65±0.14
48	CH21B	15,17-DiMeC_{32}	3343	11.60±1.24	4.97±0.18	1.33±0.11	2.94±0.13	2.24±0.41	2.73±0.51	0.75±0.13	1.99±0.07	1.96±0.12	2.78±0.18
49	CH22U	$C_{33:1}$	3390	0.31±0.07	0.18±0.10	0.29±0.11	0.45±0.14	0.25±0.01	0.66±0.11	0.21±0.01	0.84±0.31	1.19±0.41	1.52±0.03
50	CH23B	11-/13-/15-MeC_{34}	3423	0.65±0.07	1.07±0.15	0.41±0.08	0.69±0.16	0.33±0.12	0.48±0.06	0.40±0.11	1.41±0.22	1.76±0.12	1.27±0.12
51	CH24B	13,17,21-/11,15,19-TriMeC_{32}	3470	13.58±1.20	18.77±1.12	13.35±1.02	23.04±0,23	13.68±1.36	21.38±1.02	12.56±1.02	16.61±1.23	17.24±0.75	11.81±0.45
52	CH25B	13-/17-/18-/22-MeC_{35}	3524	10.14±1.02	14.75±0.16	9.88±1.32	19.99±1.68	12.42±1.02	14.52±0.25	7.90±0.62	11.67±0.12	10.47±0.63	14.12±0.21
53	CH26B	12-/14-/16-MeC_{36}	3631	1.12±0.04	1.75±0.19	1.17±0.14	2.31±0.37	1.05±0.04	1.83±0.05	0.74±0.12	1.99±0.02	1.17±0.14	2.63±0.06
54	CH27B	5,19-/5,21-/4,16-/2,14-DiMeC_{35}	3684	0.98±0.45	1.10±0.03	0.88±0.01	1.60±0.31	0.97±0.01	1.30±0.12	0.60±0.04	1.23±0.02	0.97±0.07	1.66±0.12
55	CH28B	17,19-DiMeC_{36}	3738	0.40±0.07	0.51±0.01	0.54±0.03	0.93±0.03	0.58±0.03	0.70±0.03	0.25±0.01	0.38±0.05	0.38±0.03	0.62±0.05
56	CH29B	2,10-DiMeC_{36}	3791	Tr	-	0.03±0.00	0.05±0.01	0.02±0.01	0.02±0.01	-	Tr	0.02±0.00	0.04±0.01
57	CH30B	11-/13-/15-MeC_{38}	3832	0.05±0.01	-	0.04±0.01	0.09±0.01	Tr	0.05±0.01	-	0.04±0.01	0.03±0.01	0.10±0.06
58	CH31B	15,19-DiMeC_{38}	3945	1.32±0.41	1.29±0.20	1.04±0.12	1.52±0.03	1.21±0.02	1.87±0.12	0.85±0.12	1.93±0.12	3.16±0.06	3.19±0.07
59	CH32L	*n*-Tetracontane	4001	2.72±0.23	2.13±0.15	2.94±0.33	4.45±0.36	3.54±0.12	4.49±0.33	1.73±0,02	2.33±0.13	2.42±0.05	1.96±0.07

[a] Abbreviation of the compounds corresponds with the Figs 2-4.

[b] Retention Index RI calculated for DB-5 column, Tr ≤ 0.01%.
