# Supplementary material for: Epicuticular chemistry reinforces the new taxonomic classification of the Bactrocera dorsalis species complex (Diptera: Tephritidae, Dacinae)
Source: PLoS One. 2017 Sep 5;12(9):e0184102. doi: 10.1371/journal.pone.0184102 (PMC5584755; doi:10.1371/journal.pone.0184102)
Supplement: S2 Table — Compounds are listed in order of their contributions (δi) to the average dissimilarity 5(δi) between the two groups, with a cut-off when the cumulative percent contribution (∑δi%) to δi reaches 70%. Numbering of the compounds corresponds to Fig 1 and S1 Table. (PDF) [file pone.0184102.s002.pdf]

S2 Table. A comparison of the average abundance of important fatty acid esters between five enitites of *Bacrocera* females [*B. dorsalis* (DOR), *B. carambolae* (CAR), *B. invadens* (INV), *B. papayae* (PAP), *B. philippinensis* (PHI)]. The compounds are listed in the order of their contribution (δ_i) to the average dissimilarity 5(δ_i) between the two groups, with a cut-off when the cumulative percent contribution ($\sum\delta_i$%) to δ_i reaches 70%. The numbering of the compounds corresponds to Fig 1 and S1 Table.

Compound	Abundance		δ_i	δ_i/SD(δ_i)	% contr. diss.	$\sum\delta_i$%
	PHI	PAP				
E02	0,225	0,737	0,021	3,124	0,159	0,159
E03	2,131	1,897	0,010	1,117	0,079	0,238
E07	1,854	1,687	0,010	1,440	0,074	0,313
E16	0,371	0,527	0,009	1,129	0,073	0,385
E15	1,289	1,143	0,009	1,291	0,068	0,454
E10	1,847	1,776	0,009	1,320	0,067	0,521
E14	0,181	0,284	0,008	1,598	0,061	0,582
E11	1,807	1,674	0,007	0,985	0,054	0,636
E04	0,185	0,345	0,007	1,515	0,054	0,690
E12	0,231	0,246	0,007	1,946	0,053	0,743
E06	0,332	0,380	0,005	1,568	0,038	0,781
E08	0,385	0,427	0,005	1,495	0,037	0,818
E05	0,076	0,162	0,004	1,447	0,030	0,848
E22	0,452	0,389	0,004	1,194	0,029	0,877
E01	0,194	0,120	0,004	1,635	0,029	0,906
E09	0,307	0,278	0,004	1,805	0,028	0,933
E17	0,308	0,228	0,003	1,368	0,024	0,957
E18	0,139	0,103	0,002	1,596	0,013	0,970
E20	0,129	0,101	0,002	1,505	0,013	0,984
E19	0,027	0,002	0,001	2,196	0,008	0,991
E13	0,025	0,013	0,001	1,736	0,006	0,997
E21	0,022	0,012	0,000	1,181	0,003	1,000
Compound	Abundance		δ_i	δ_i/SD(δ_i)	% contr. diss.	$\sum\delta_i$%
	PHI	INV				
E02	0,225	0,700	0,018	2,693	0,113	0,113
E01	0,194	0,532	0,013	1,933	0,080	0,194
E07	1,854	2,070	0,011	1,454	0,070	0,263
E06	0,332	0,619	0,011	2,859	0,068	0,332

E10	1,847	1,880	0,010	2,577	0,061	0,393
E16	0,371	0,605	0,010	1,070	0,061	0,454
E11	1,807	1,940	0,010	1,981	0,061	0,514
E12	0,231	0,390	0,009	1,649	0,058	0,572
E03	2,131	2,060	0,009	1,082	0,058	0,630
E09	0,307	0,525	0,008	2,579	0,052	0,682
E15	1,289	1,260	0,008	1,703	0,048	0,730
E22	0,452	0,287	0,007	1,734	0,047	0,777
E14	0,181	0,000	0,007	0,951	0,045	0,822
E04	0,185	0,348	0,007	1,508	0,042	0,864
E08	0,385	0,501	0,006	1,775	0,038	0,903
E17	0,308	0,180	0,005	1,755	0,031	0,933
E05	0,076	0,133	0,003	1,740	0,020	0,953
E20	0,129	0,106	0,003	1,943	0,016	0,969
E18	0,139	0,106	0,002	1,911	0,015	0,984
E13	0,025	0,044	0,001	1,395	0,008	0,992
E19	0,027	0,027	0,001	1,609	0,004	0,996
E21	0,022	0,016	0,001	1,766	0,004	1,000

**Compound**	**Abundance**		**δ_i**	**$\delta_i/SD(\delta_i)$**	**% contr. diss.**	**$\sum\delta_i\%$**
	**PHI**	**DOR**				
E03	2,131	2,056	0,020	1,549	0,125	0,125
E07	1,854	2,014	0,016	1,555	0,102	0,227
E12	0,231	0,571	0,014	1,818	0,086	0,313
E01	0,194	0,555	0,013	2,114	0,083	0,396
E11	1,807	1,910	0,013	1,428	0,080	0,476
E14	0,181	0,433	0,011	1,496	0,071	0,547
E10	1,847	1,943	0,011	1,357	0,068	0,615
E16	0,371	0,423	0,011	1,079	0,068	0,683
E15	1,289	1,351	0,009	1,478	0,059	0,741
E02	0,225	0,424	0,008	1,285	0,049	0,790
E06	0,332	0,511	0,007	1,207	0,041	0,831
E04	0,185	0,214	0,005	1,520	0,033	0,864
E22	0,452	0,521	0,005	1,413	0,031	0,895
E08	0,385	0,354	0,004	1,022	0,024	0,918
E09	0,307	0,364	0,004	1,186	0,023	0,941
E17	0,308	0,292	0,003	1,375	0,017	0,958
E05	0,076	0,014	0,002	1,066	0,015	0,972
E20	0,129	0,109	0,001	1,254	0,009	0,981
E18	0,139	0,133	0,001	1,552	0,009	0,990
E19	0,027	0,014	0,001	1,766	0,005	0,995
E13	0,025	0,013	0,000	1,378	0,003	0,998

E21	0,022	0,025	0,000	1,335	0,002	1,000
**Compound**	**Abundance**		**δ_i**	**δ_i/SD(δ_i)**	**% contr. diss.**	**$\sum\delta_i$%**
	**PHI**	**CAR**				
E04	0,185	0,529	0,014	2,341	0,095	0,095
E07	1,854	2,034	0,013	1,470	0,089	0,185
E10	1,847	2,000	0,012	1,463	0,085	0,270
E02	0,225	0,513	0,011	2,176	0,078	0,348
E11	1,807	1,668	0,010	1,338	0,071	0,418
E03	2,131	2,152	0,010	1,605	0,070	0,489
E12	0,231	0,347	0,009	1,212	0,065	0,553
E16	0,371	0,539	0,009	1,118	0,063	0,616
E15	1,289	1,237	0,009	1,464	0,060	0,677
E06	0,332	0,510	0,007	1,169	0,047	0,723
E08	0,385	0,492	0,007	1,405	0,046	0,770
E14	0,181	0,076	0,006	0,990	0,044	0,814
E22	0,452	0,561	0,006	1,294	0,044	0,858
E05	0,076	0,153	0,004	1,468	0,029	0,887
E17	0,308	0,211	0,004	1,259	0,029	0,916
E01	0,194	0,247	0,004	1,226	0,025	0,941
E09	0,307	0,318	0,002	0,977	0,017	0,958
E18	0,139	0,121	0,002	1,396	0,014	0,973
E20	0,129	0,110	0,002	1,542	0,014	0,987
E19	0,027	0,009	0,001	1,806	0,006	0,992
E13	0,025	0,009	0,001	1,379	0,004	0,996
E21	0,022	0,008	0,001	1,569	0,004	1,000
**Compound**	**Abundance**		**δ_i**	**δ_i/SD(δ_i)**	**% contr. diss.**	**$\sum\delta_i$%**
	**PAP**	**INV**				
E01	0,120	0,532	0,015	2,955	0,123	0,123
E07	1,687	2,070	0,014	3,732	0,114	0,237
E14	0,284	0,000	0,011	2,247	0,085	0,322
E11	1,674	1,940	0,010	2,544	0,079	0,401
E09	0,278	0,525	0,009	2,688	0,074	0,475
E06	0,380	0,619	0,009	1,565	0,072	0,547
E03	1,897	2,060	0,008	1,121	0,065	0,612
E16	0,527	0,605	0,006	1,444	0,048	0,660
E22	0,389	0,287	0,006	2,078	0,045	0,704
E15	1,143	1,260	0,005	1,308	0,043	0,747
E12	0,246	0,390	0,005	0,929	0,043	0,790
E10	1,776	1,880	0,005	1,555	0,039	0,829
E02	0,737	0,700	0,005	1,231	0,037	0,866
E08	0,427	0,501	0,004	1,218	0,031	0,896

E17	0,228	0,180	0,003	1,995	0,021	0,917
E20	0,101	0,106	0,002	1,446	0,018	0,935
E18	0,103	0,106	0,002	1,419	0,015	0,950
E04	0,345	0,348	0,002	1,193	0,014	0,964
E05	0,162	0,133	0,002	1,625	0,013	0,977
E13	0,013	0,044	0,001	1,162	0,012	0,989
E19	0,002	0,027	0,001	1,458	0,008	0,996
E21	0,012	0,016	0,000	1,409	0,004	1,000

**Compound**	**Abundance**		δ_i	$\delta_i/SD(\delta_i)$	**% contr. diss.**	$\sum\delta_i\%$
	**PAP**	**DOR**				
E03	1,897	2,056	0,020	2,429	0,119	0,119
E07	1,687	2,014	0,017	2,338	0,105	0,224
E01	0,120	0,555	0,016	3,493	0,096	0,320
E11	1,674	1,910	0,014	3,817	0,084	0,404
E02	0,737	0,424	0,012	1,625	0,075	0,479
E12	0,246	0,571	0,012	1,864	0,070	0,549
E10	1,776	1,943	0,009	2,144	0,056	0,605
E15	1,143	1,351	0,009	1,759	0,056	0,661
E16	0,527	0,423	0,009	1,151	0,056	0,717
E14	0,284	0,433	0,009	1,251	0,052	0,769
E06	0,380	0,511	0,007	1,331	0,041	0,810
E04	0,345	0,214	0,006	0,991	0,034	0,844
E05	0,162	0,014	0,006	7,215	0,034	0,877
E22	0,389	0,521	0,005	1,571	0,030	0,907
E09	0,278	0,364	0,004	1,289	0,026	0,934
E08	0,427	0,354	0,004	1,220	0,025	0,959
E17	0,228	0,292	0,003	1,541	0,015	0,974
E18	0,103	0,133	0,001	1,429	0,009	0,983
E20	0,101	0,109	0,001	1,358	0,007	0,990
E19	0,002	0,014	0,001	0,751	0,004	0,994
E13	0,013	0,013	0,001	1,868	0,004	0,997
E21	0,012	0,025	0,000	1,504	0,003	1,000

**Compound**	**Abundance**		δ_i	$\delta_i/SD(\delta_i)$	**% contr. diss.**	$\sum\delta_i\%$
	**PAP**	**CAR**				
E07	1,687	2,034	0,013	1,468	0,109	0,109
E03	1,897	2,152	0,010	1,763	0,087	0,196
E10	1,776	2,000	0,009	1,160	0,076	0,272
E02	0,737	0,513	0,009	1,740	0,072	0,345
E14	0,284	0,076	0,008	1,453	0,066	0,411
E12	0,246	0,347	0,007	1,140	0,060	0,471
E04	0,345	0,529	0,007	2,591	0,059	0,530

E11	1,674	1,668	0,007	2,349	0,058	0,588
E06	0,380	0,510	0,007	1,300	0,056	0,644
E22	0,389	0,561	0,007	1,340	0,056	0,700
E15	1,143	1,237	0,006	1,458	0,052	0,752
E08	0,427	0,492	0,005	1,448	0,045	0,797
E16	0,527	0,539	0,005	1,216	0,043	0,840
E01	0,120	0,247	0,005	2,284	0,041	0,881
E09	0,278	0,318	0,004	1,691	0,030	0,911
E05	0,162	0,153	0,003	3,299	0,027	0,938
E17	0,228	0,211	0,002	1,273	0,019	0,957
E18	0,103	0,121	0,002	1,909	0,018	0,975
E20	0,101	0,110	0,002	1,669	0,015	0,990
E13	0,013	0,009	0,001	1,418	0,005	0,995
E19	0,002	0,009	0,000	0,835	0,003	0,998
E21	0,012	0,008	0,000	2,117	0,003	1,000
**Compound**	**Abundance**		**δ_i**	**$\delta_i/SD(\delta_i)$**	**% contr. diss.**	**$\sum\delta_i$%**
	**INV**	**DOR**				
E03	2,060	2,056	0,018	1,622	0,119	0,119
E14	0,000	0,433	0,015	1,943	0,096	0,215
E07	2,070	2,014	0,013	1,331	0,086	0,301
E11	1,940	1,910	0,011	1,496	0,071	0,372
E02	0,700	0,424	0,010	1,437	0,068	0,440
E16	0,605	0,423	0,009	1,195	0,062	0,502
E22	0,287	0,521	0,009	1,891	0,056	0,558
E12	0,390	0,571	0,008	1,471	0,052	0,610
E10	1,880	1,943	0,008	2,090	0,051	0,661
E15	1,260	1,351	0,007	1,995	0,044	0,705
E06	0,619	0,511	0,006	1,689	0,042	0,747
E01	0,532	0,555	0,006	1,530	0,040	0,787
E09	0,525	0,364	0,006	1,428	0,040	0,826
E08	0,501	0,354	0,006	1,462	0,038	0,864
E04	0,348	0,214	0,005	0,985	0,035	0,899
E17	0,180	0,292	0,004	1,952	0,027	0,926
E05	0,133	0,014	0,004	2,858	0,027	0,953
E20	0,106	0,109	0,002	1,843	0,014	0,968
E18	0,106	0,133	0,002	1,909	0,014	0,981
E13	0,044	0,013	0,001	1,293	0,009	0,991
E19	0,027	0,014	0,001	1,750	0,006	0,996
E21	0,016	0,025	0,001	1,684	0,004	1,000
**Compound**	**Abundance**		**δ_i**	**$\delta_i/SD(\delta_i)$**	**% contr. diss.**	**$\sum\delta_i$%**
	**INV**	**CAR**				

E11	1,940	1,668	0,011	1,527	0,091	0,091
E22	0,287	0,561	0,010	1,603	0,084	0,175
E01	0,532	0,247	0,010	1,955	0,083	0,258
E12	0,390	0,347	0,008	1,642	0,069	0,327
E07	2,070	2,034	0,008	1,676	0,068	0,394
E03	2,060	2,152	0,008	1,307	0,063	0,458
E09	0,525	0,318	0,007	2,380	0,062	0,520
E10	1,880	2,000	0,007	1,195	0,060	0,581
E02	0,700	0,513	0,007	1,282	0,056	0,637
E04	0,348	0,529	0,006	2,521	0,054	0,690
E06	0,619	0,510	0,006	1,835	0,052	0,742
E16	0,605	0,539	0,005	1,351	0,044	0,786
E15	1,260	1,237	0,005	1,803	0,043	0,829
E08	0,501	0,492	0,005	1,562	0,041	0,870
E17	0,180	0,211	0,003	1,437	0,023	0,893
E05	0,133	0,153	0,003	1,323	0,023	0,916
E14	0,000	0,076	0,003	1,309	0,022	0,938
E20	0,106	0,110	0,002	1,547	0,020	0,958
E18	0,106	0,121	0,002	1,462	0,019	0,977
E13	0,044	0,009	0,001	1,263	0,012	0,989
E19	0,027	0,009	0,001	1,439	0,007	0,996
E21	0,016	0,008	0,001	1,241	0,004	1,000

**Compound**	**Abundance**		**δ_i**	**$\delta_i/SD(\delta_i)$**	**% contr. diss.**	**$\sum\delta_i\%$**
	**DOR**	**CAR**				
E03	2,056	2,152	0,018	1,325	0,112	0,112
E07	2,014	2,034	0,015	1,464	0,096	0,209
E11	1,910	1,668	0,013	1,521	0,086	0,294
E14	0,433	0,076	0,012	1,471	0,077	0,371
E04	0,214	0,529	0,012	1,817	0,076	0,447
E12	0,571	0,347	0,011	1,662	0,071	0,518
E01	0,555	0,247	0,011	2,127	0,067	0,586
E10	1,943	2,000	0,010	1,492	0,064	0,649
E16	0,423	0,539	0,009	1,169	0,056	0,705
E15	1,351	1,237	0,008	1,409	0,048	0,754
E08	0,354	0,492	0,006	1,272	0,039	0,793
E02	0,424	0,513	0,006	1,979	0,039	0,832
E06	0,511	0,510	0,005	0,999	0,033	0,865
E05	0,014	0,153	0,005	1,579	0,030	0,896
E22	0,521	0,561	0,005	1,203	0,030	0,925
E17	0,292	0,211	0,004	1,369	0,022	0,947
E09	0,364	0,318	0,003	1,126	0,021	0,969

E18	0,133	0,121	0,002	1,237	0,012	0,980
E20	0,109	0,110	0,002	1,509	0,010	0,991
E19	0,014	0,009	0,001	0,950	0,004	0,995
E21	0,025	0,008	0,001	1,991	0,004	0,998
E13	0,013	0,009	0,000	1,580	0,002	1,000
