# Supplementary figures and images for: Epicuticular chemistry reinforces the new taxonomic classification of the Bactrocera dorsalis species complex (Diptera: Tephritidae, Dacinae)
Source: PLoS One. 2017 Sep 5;12(9):e0184102. doi: 10.1371/journal.pone.0184102 (PMC5584755; doi:10.1371/journal.pone.0184102)

Female

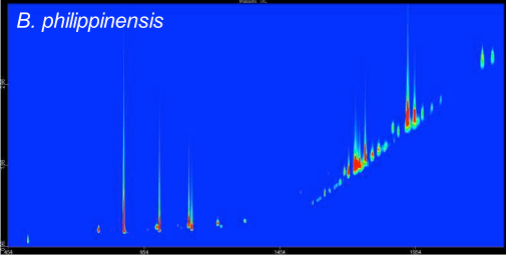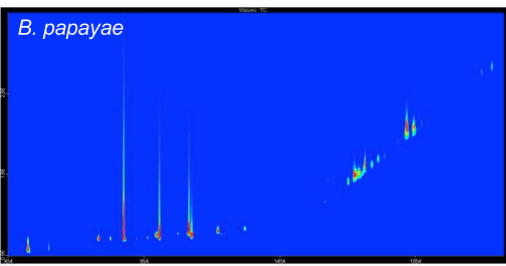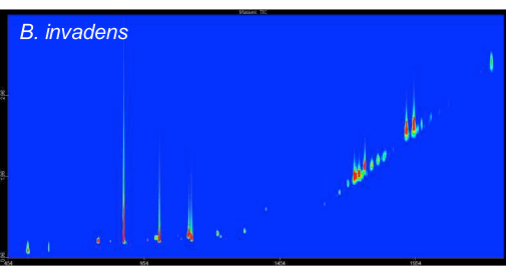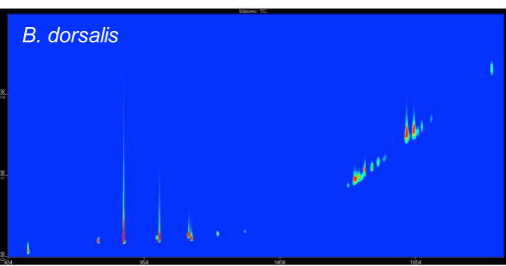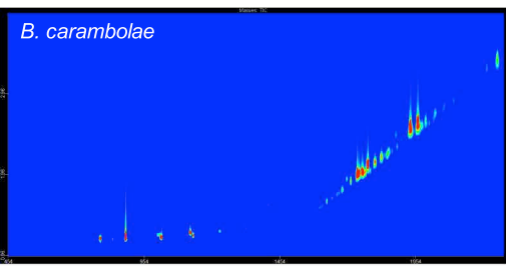

Male

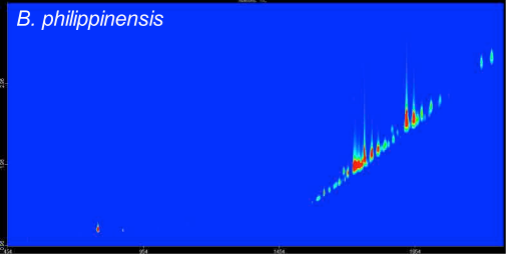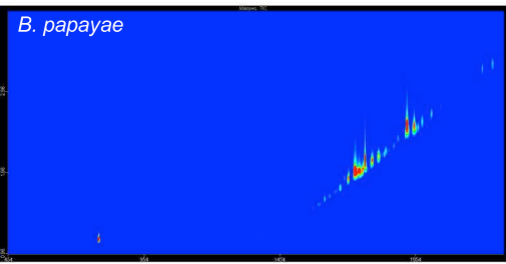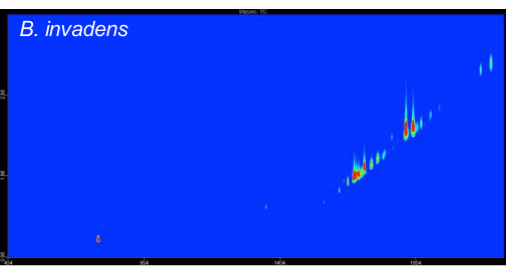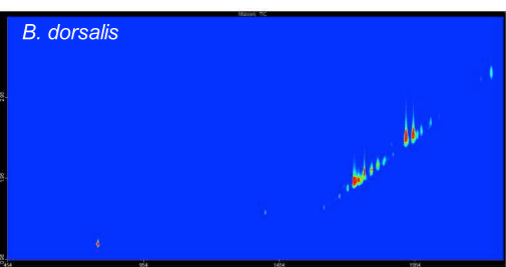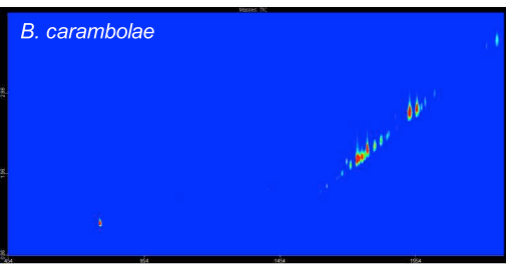

Supplement: S1 Fig — Cuticular profiles of female (A) and male (B). Intensity of the signals is colour coded from blue (zero) to red (maximum). (PDF) [file pone.0184102.s003.pdf]

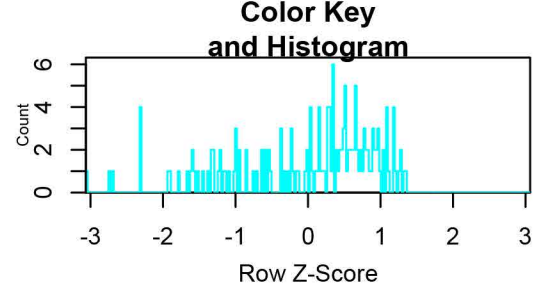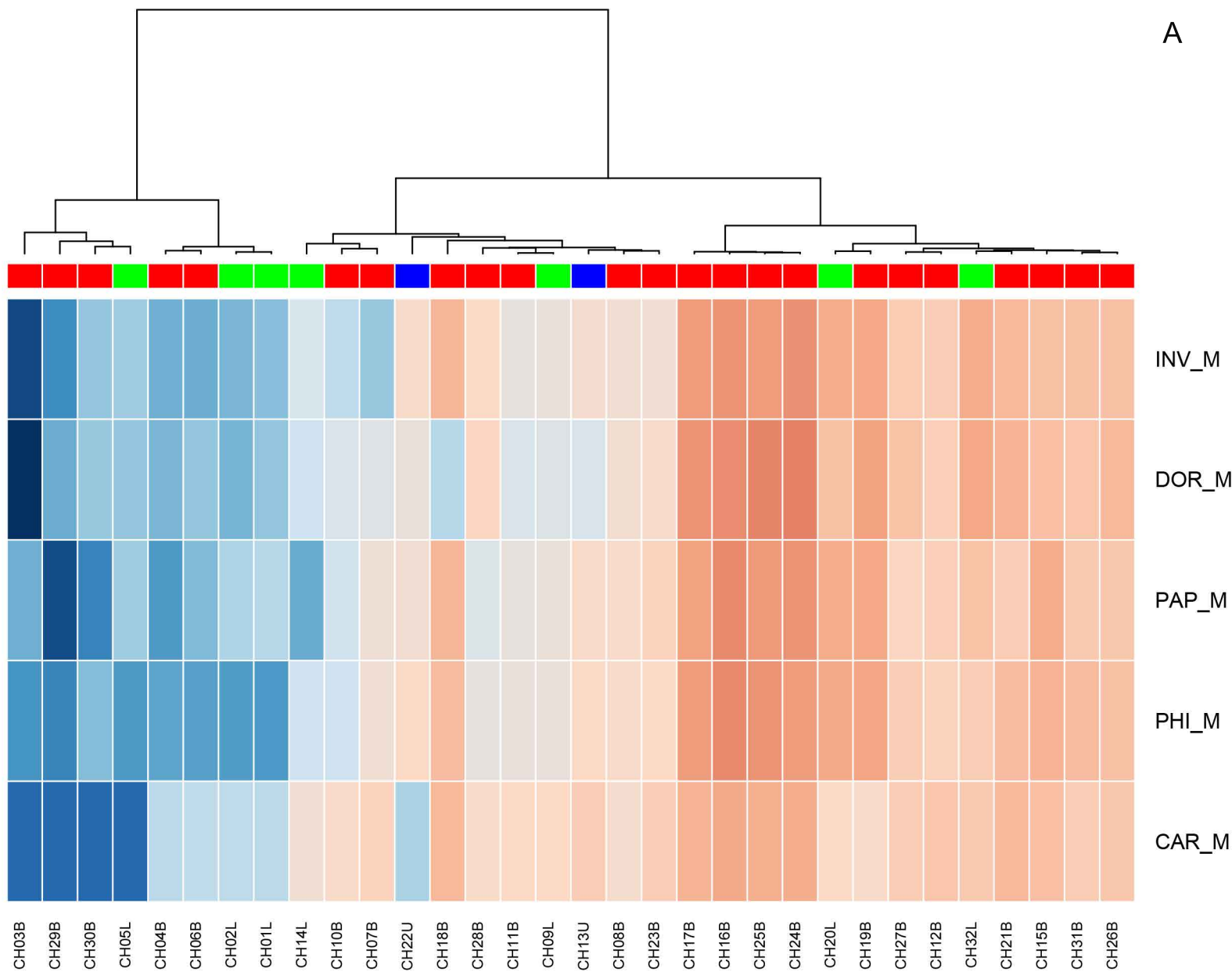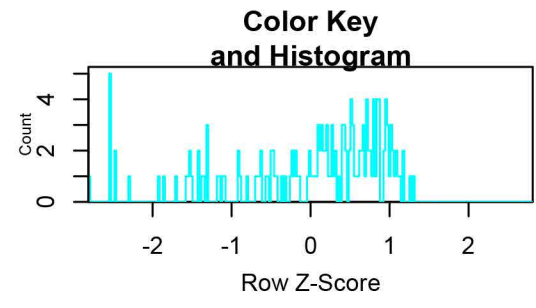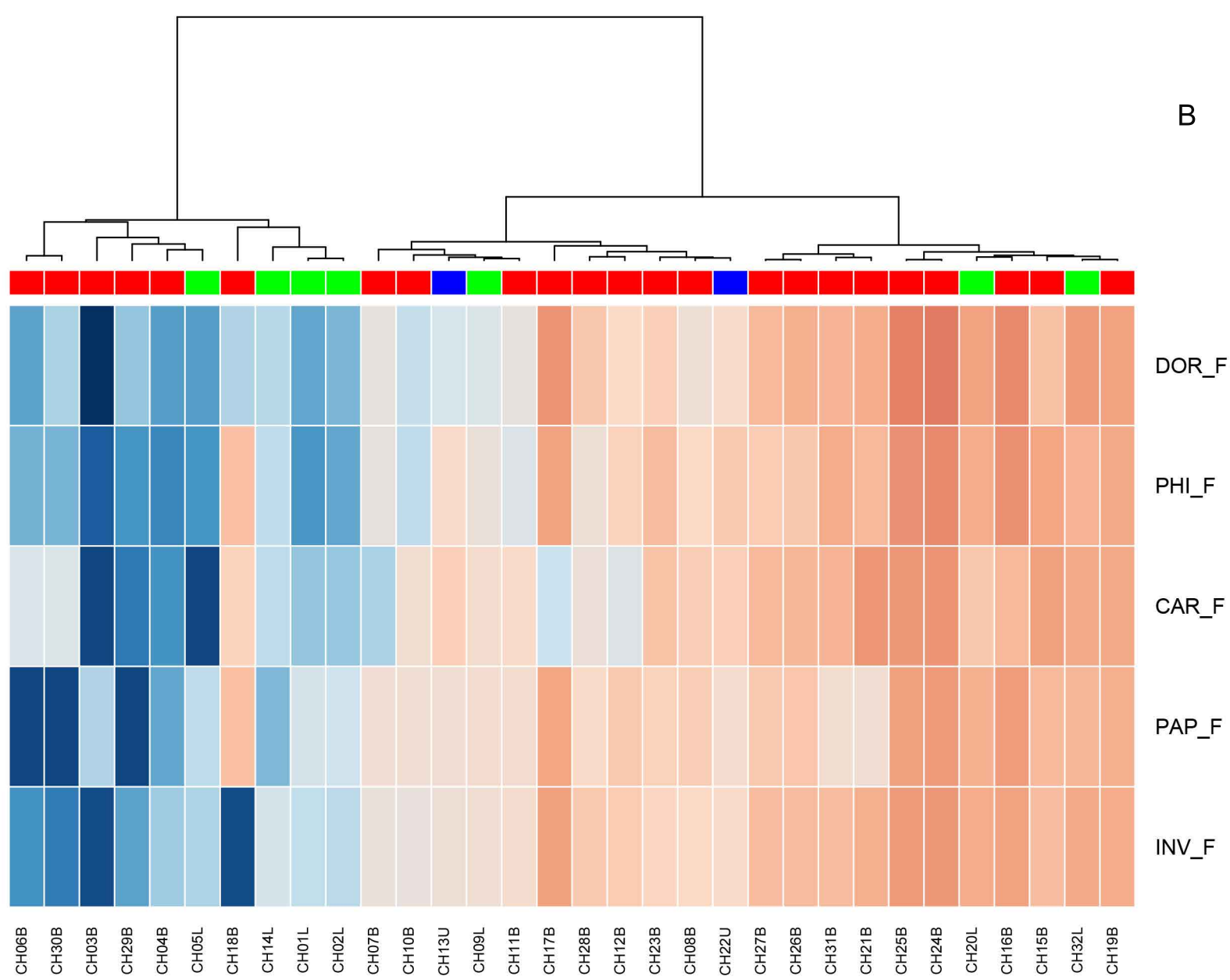

Supplement: S2 Fig — (A) male, (B) female. DOR = B. dorsalis, CAR = B. carambolae, INV = B. ‘syn. invadens’, PAP = B. ‘syn. papayae’, PHI = B. ‘syn. philippinensis’). Columns are colour coded according to chemical classes (violet CHL = linear hydrocarbon, red CHB = methyl-branched hydrocarbon, green CHU = unsaturated hydrocarbon). Dendrograms are created using correlation-based distances and the Ward method of hierarchical clustering (P < 0.05). Compounds are assigned according to S1 Table. (PDF) [file pone.0184102.s004.pdf]

A

# Hierarchical clustering on the factor map

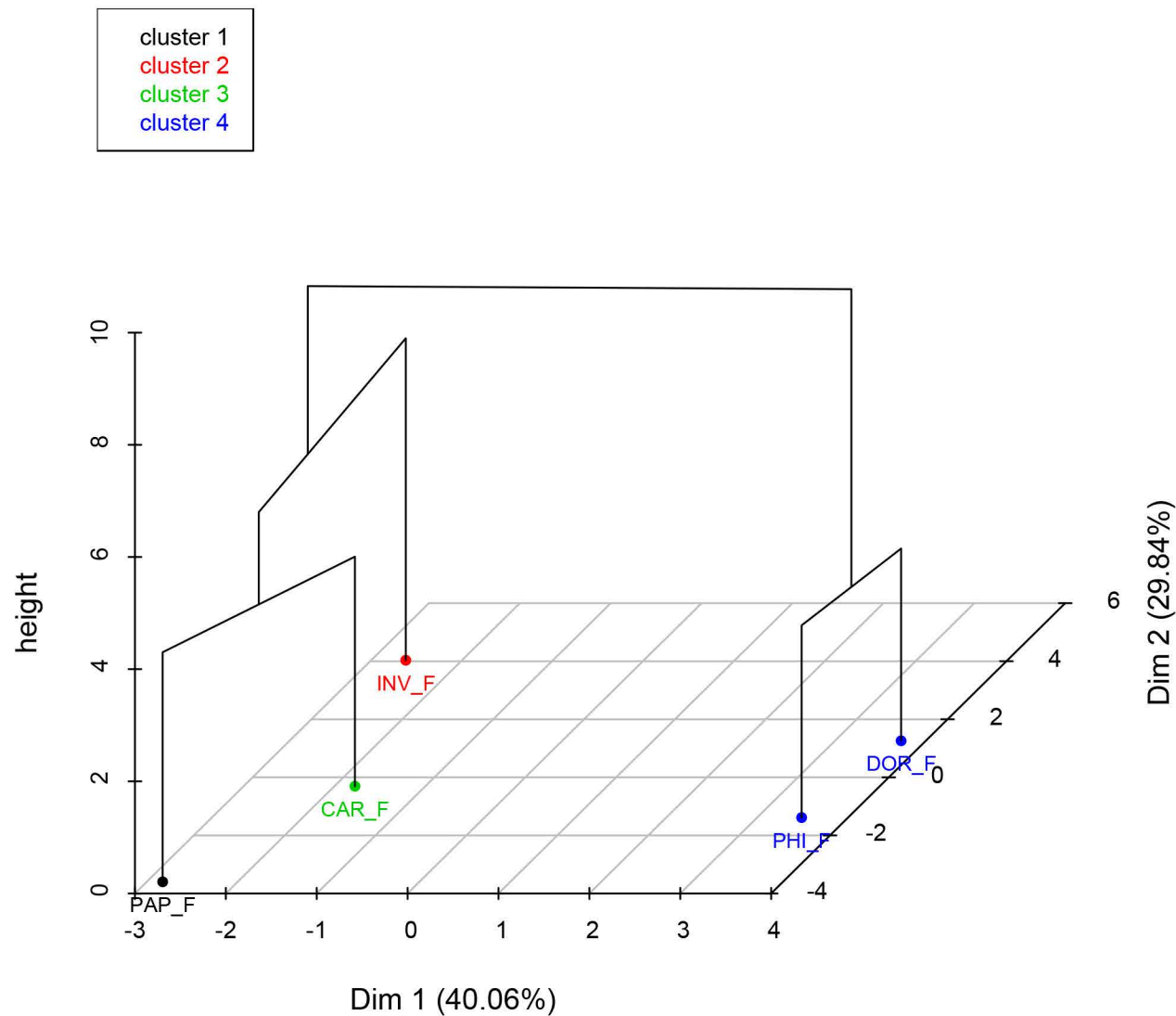

B

# Variables factor map (PCA)

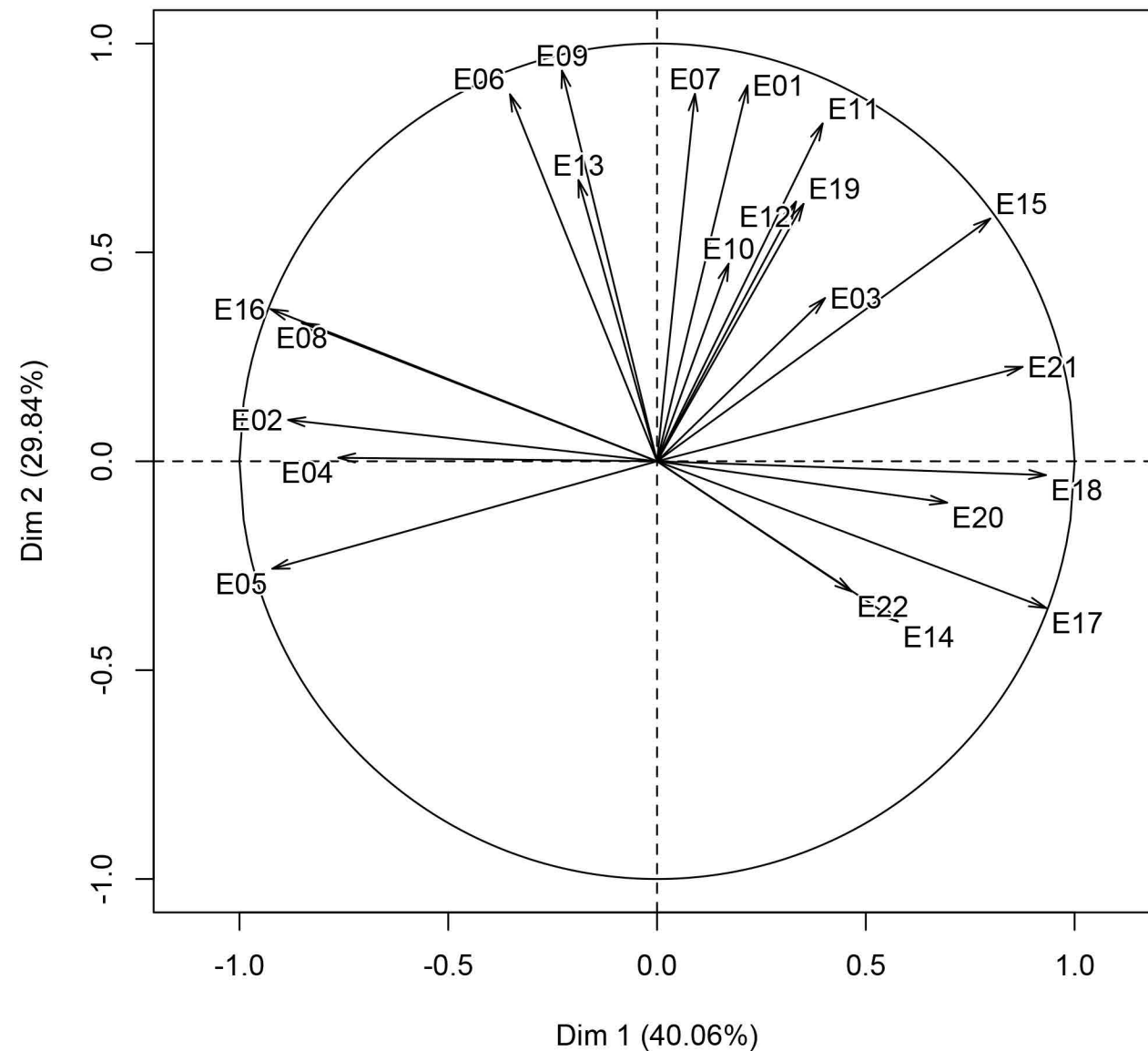

Supplement: S3 Fig — E01–22 were identified on the cuticle of five Bactrocera entities (DOR = B. dorsalis, CAR = B. carambolae, INV = B. ‘syn. invadens’, PAP = B. ‘syn. papayae’, PHI = B. ‘syn. philippinensis’). (A) Variables factor map represents projection of variables on plane defined by the first two principal components. (B) Hierarchical clustering is score plot describing the species and their clustering. Colours indicate particular clusters. (PDF) [file pone.0184102.s005.pdf]
